# Murine splenic B cells express corticotropin-releasing hormone receptor 2 that affect their viability during a stress response

**DOI:** 10.1038/s41598-017-18401-y

**Published:** 2018-01-09

**Authors:** Guillaume Harlé, Sandra Kaminski, David Dubayle, Jean-Pol Frippiat, Armelle Ropars

**Affiliations:** 1 EA 7300, Stress Immunity Pathogens Laboratory, Faculty of Medicine, Lorraine University, Vandoeuvre-lès-Nancy, France; 2 CNRS UMR 8119, Centre de neurophysique, physiologie et pathologie, University of Paris Descartes, Paris, France

## Abstract

Chronic stress is now recognized as a risk factor for disease development and/or exacerbation. It has been shown to affect negatively the immune system and notably the humoral immune response. Corticotropin-releasing hormone (CRH) is known to play a crucial role in stress response. CRH receptors are expressed on different immune cells such as granulocytes, monocytes and T cells. However, up to now, no CRH receptor has been described on B cells which are key players of the humoral immune response. In order to highlight new pathways by which stress may impact immunity, we investigated the role of CRH in B cells. Here we show that splenic B cells express the CRH receptor 2 (CRHR2), but not CRHR1. This receptor is functional since CRH treatment of B cells activates different signaling pathways (e.g. p38) and decreases B cell viability. Finally, we show that immunization of mice with two types of antigens induces a more intense CRHR staining in secondary lymphoid organs where B cells are known to respond to the antigen. Altogether our results demonstrate, for the first time, that CRH is able to modulate directly B cell activity through the presence of CRHR2.

## Introduction

Stress is known to impact the immune system. Effects depend on the duration, the intensity and the type of stressor. Many studies have demonstrated that acute/short-term stress could favor immune responses while chronic/long-term stress could alter them^1,2^. Chronic stress is a risk factor for developing and/or exacerbating depressive disorders, inflammatory diseases, infections, cancers and depressive disorders^3,4^. Indeed, chronic stress has been shown to affect different immune cell functions such as natural killer (NK) cell activity, T and B cells populations and proliferation, antibody production as well as immune response to vaccines^3^.

Corticotropin-releasing hormone (CRH), a 41 amino acid peptide produced essentially by the hypothalamus, is the main mediator of the stress effects on the hypothalamic-pituitary-adrenocortical axis (HPA)^5^. Indeed, in cases of stress, CRH production increases and activates the HPA axis which in turn stimulates the anterior pituitary to increase adrenocorticotrophic hormone (ACTH) synthesis^6,7^. In response to ACTH, adrenal glands produce catecholamines and glucocorticoids. Catecholamines will activate the sympathetic nervous system while glucocorticoids will restrain inflammatory mediators action and protect the organism from the onset of an exaggerated inflammatory response^8,9^. Nevertheless, the role of CRH is not restricted to the central nervous system (CNS). Indeed, hypothalamic CRH can cross the blood-brain barrier and act in the periphery^10^. CRH receptors (CRHR1 and CRHR2) are not only present in the CNS but also in various tissues such as the skin, adrenal glands, heart, spleen and thymus^11–14^. Blood immune cells such as granulocytes, monocytes or T cells also express CRHR^15,16^. Furthermore, all these tissues and cell types are able to produce small amounts of CRH^11,14,17,18^.

*In vitro* studies have demonstrated that CRH is able to activate cAMP and to modify cytokine production. Indeed, CRH increases IL-1, IL-2 and IL-6, and reduces IFNγ production by human blood mononuclear cells^19–23^. CRH induces the proliferation of human blood T cells and increases their IL-2 receptor membrane expression^24^. Administration of CRH, either intracerebroventricularly or intravenously, reduces splenic NK cytotoxicity as well as lymphocyte proliferation^25,26^. Labeur *et al*. have shown that long-term infusion of CRH into the lateral ventricle of rats reduced thymus and spleen weight and reduced *in vitro* splenic T and B cell proliferation^27^.

B cells are key players of humoral immunity through their ability to produce antibodies and enhance antibody affinity *via* somatic hypermutation^28^. This latter phenomenon contributes to a better protection of the organism. Depending on the nature of the antigen (T cell-dependent or T cell-independent), B cells require or not cooperation with T cells to mount their response. As T cells express CRHR, CRH can affect this cell type and consequently B cell responses in the case of T cell-dependent antigens (indirect action). However, it is also of crucial interest to determine if B cells can be directly affected by CRH. Some studies have tried to address this question but conflicting results were reported. Using human blood mononuclear cells, Leu and Singh showed that CRH inhibits antibody production while Smith *et al*., using murine splenocytes, showed that CRH enhances antigen-specific antibody production^29,30^. CRH microinjection into the lateral ventricle of rats was shown to slow down antibody induction in response to a T cell-dependent antigen both after primary or secondary immunization^31^. When CRH was injected intraperitoneally or subcutaneously, this diminution did not occur. Finally, it was shown that immunization of CRH transgenic (CRH-Tg) mice decreased humoral immune response and germinal center formation^32,33^. Taken together, these studies strongly suggest that CRH is able to modulate B cell activity. However, the presence of CRH receptors on these cells has never been reported. Thus, it is not known if CRH can acts directly on B cells.

As a first step to determine whether CRH action on B cells could be direct, we searched for CRHR on purified splenic murine B cells and discovered that these cells express CRHR2 but not CRHR1. These CRH receptors are functional as the addition of CRH to splenic B cells activates p38β, p38δ MAP kinases, GSK3β phosphorylation and the PKA/CREB signaling pathway. Secondly, we were interested in the CRH effect on splenocyte viability. In the presence of the hormone, splenic B cell viability was decreased while the one of T cell remained unchanged. Finally, we showed that immunizations led to a more specific CRHR labeling in secondary lymphoid organs correlating with B cell areas labeling. Taken together, these data show that CRH can act directly on B cells and affect their physiology.

## Results

### Murine splenic B cells express CRHR2

**Figure 1:**
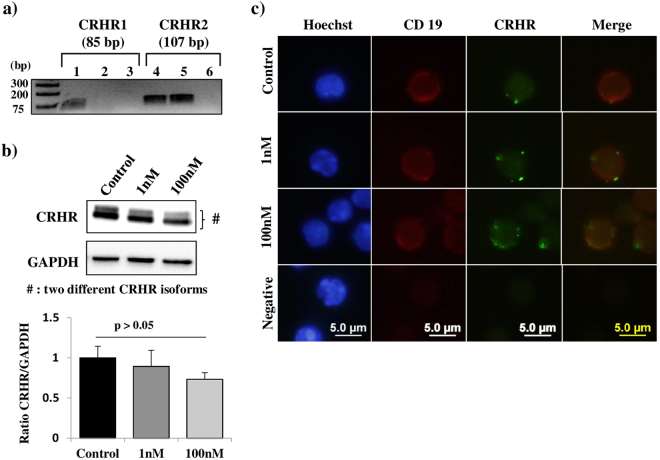
Murine splenic B cells express CRHR2 receptors. Splenocytes were incubated or not with CRH 1 or 100 nM for 48 h. Then, B cells were isolated by negative selection. (**a**) Total RNA extracted from hippocampus (positive control) and murine splenic B cells was reverse transcribed. Then, PCR amplifications were carried out using CRHR1 or CRHR2 specific primer pairs. Lanes 1 and 4: CRHR1 and CRHR2 amplification products obtained from hippocampus total RNA (positive controls). Lanes 2 and 5: amplification products obtained from splenic B cell total RNA. Lanes 3 and 6: negative controls (no cDNA). Full-length gel is shown in Supplementary Fig. S1. (**b**) Western blotting analyses. B cell protein lysates were loaded on a SDS-page gel, submitted to electrophoresis and transferred onto a PVDF membrane. The membrane was incubated with an anti-CRHR antibody, stripped and rehybridized with an anti-GAPDH antibody. Panels show bands revealed by both antibodies on the same membrane. Full-length blots are shown in Supplementary Fig. S1. (**c**) Immunofluorescence analysis of isolated splenic B cells labeled with anti-CD19 (red) and anti-CRHR (green) antibodies. Panels (a), (c) and (d) are representative of three independent experiments.

The presence of CRH receptors has been reported on murine splenic T cells and macrophages but not on splenic B cells. Thus, we investigated the presence of CRH receptors on B cells. As two types of CRH receptors have been described, CRHR1 and CRHR2, we wondered which one could be expressed by murine splenic B cells. To address this question, B cells were purified by negative selection from murine splenocytes and purity, determined by flow cytometry, was comprised between 92 and 97%. Then, RNA was extracted from these splenic B cells and RT-PCRs were performed with two pairs of primers defined in specific parts of CRHR1 and CRHR2 transcripts. As shown in Fig. 1a, no amplification was obtained with CRHR1 primers even after 40 cycles of amplification whereas amplicons were obtained with both pairs of CRHR2 oligonucleotides. Sequencing of these PCR products confirmed that amplicons were CRHR2 cDNA fragments demonstrating that murine B cells express only these receptor transcripts.

Then, we investigated whether CRHR2 protein expression level could be modulated by CRH. To address this question, splenocytes were cultured for 48 h with CRH 1 or 100 nM. As for RT-PCR experiments, B cells were purified by negative selection and the expression of CRHR2 was assessed by western blotting. Figure 1b shows that B cells express CRHR2 independently of the CRH treatment. Interestingly, two bands were observed and might correspond to different CRHR2 isoforms. To confirm CRHR2 expression by murine splenic B cells, splenocytes were cultured with 1 or 100 nM of CRH for 48 h and analyzed by immunofluorescence. As shown in Fig. 1c, CRHR2 was found to be expressed on B cells (CD19^+^). Altogether these results firmly demonstrate that splenic B cells express CRH receptors and more specifically CRHR2.

### CRH activates different signaling pathways in murine splenic B cells

**Figure 2:**
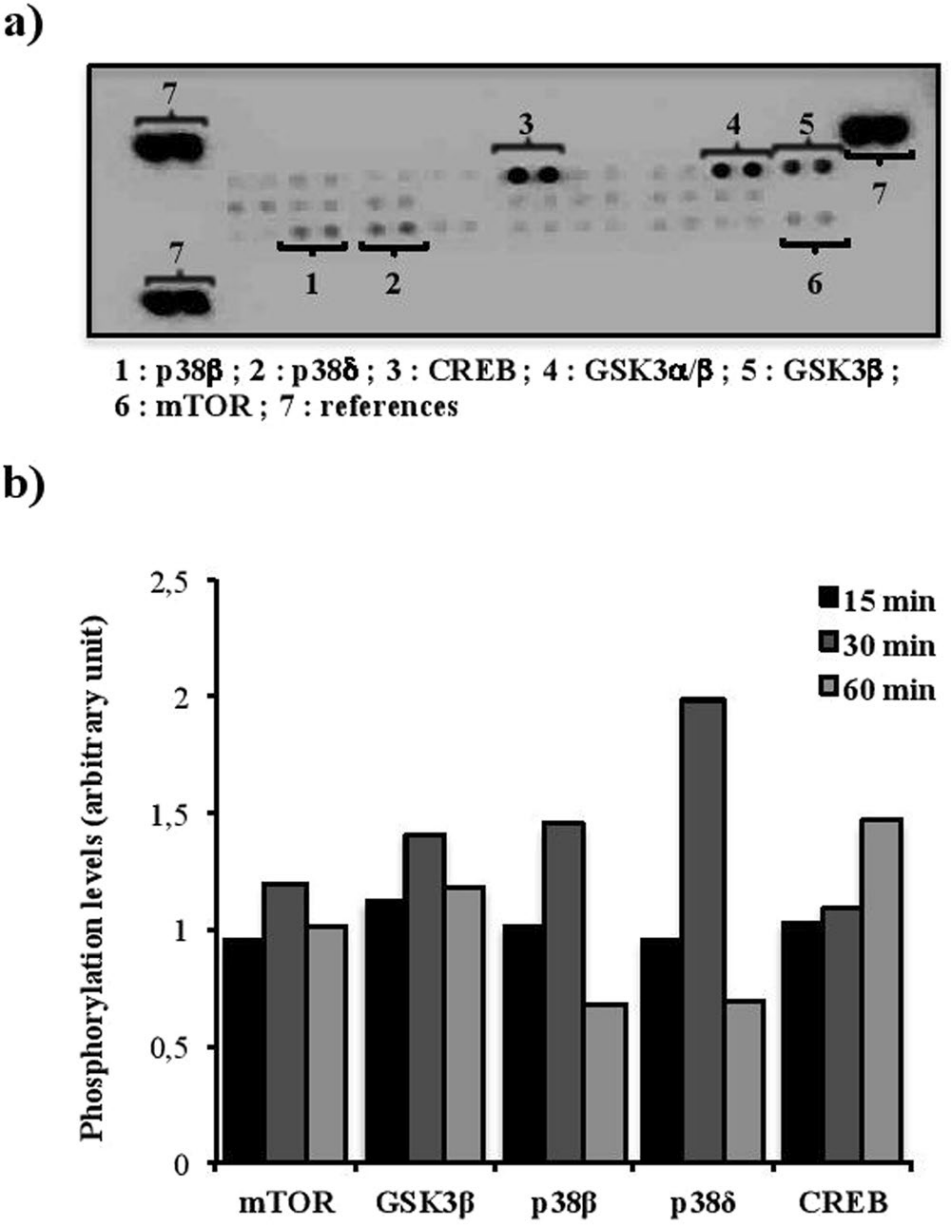
CRH activates different signaling pathways in splenic murine B cells. B lymphocytes were isolated from mice spleens by negative selection and cultured with CRH 100 nM for 15, 30 or 60 minutes. Then, cell lysates were loaded onto proteome phospho-MAPK array. (**a**) Example of obtained results. (**b**) Phosphorylation levels of the different proteins revealed by the proteome phospho-MAPK array.

To determine if CRHR2 are functional, splenic B cells were purified and incubated with CRH 100 nM for different durations (15, 30 or 60 min). Proteins were then extracted and applied onto a proteome phospho-MAPK array to screen signaling transduction pathways that can be activated by this hormone. No activation was noted after 15 min of incubation with CRH (Fig. 2). After 30 min, an increase of the phosphorylation levels of mTOR (≈ 20%), GSK3β and p38β (≈45%), and p38δ (≈100%) was observed in B cells treated with CRH compared to non CRH-treated B cells. Moreover, the phosphorylation level of CREB was increased by approximately 45% after 60 min of CRH incubation. These results indicate that different signaling pathways could be activated by CRH and that CRHR2 are functional in B cells.

### CRH affects murine splenic B cell viability

**Figure 3:**
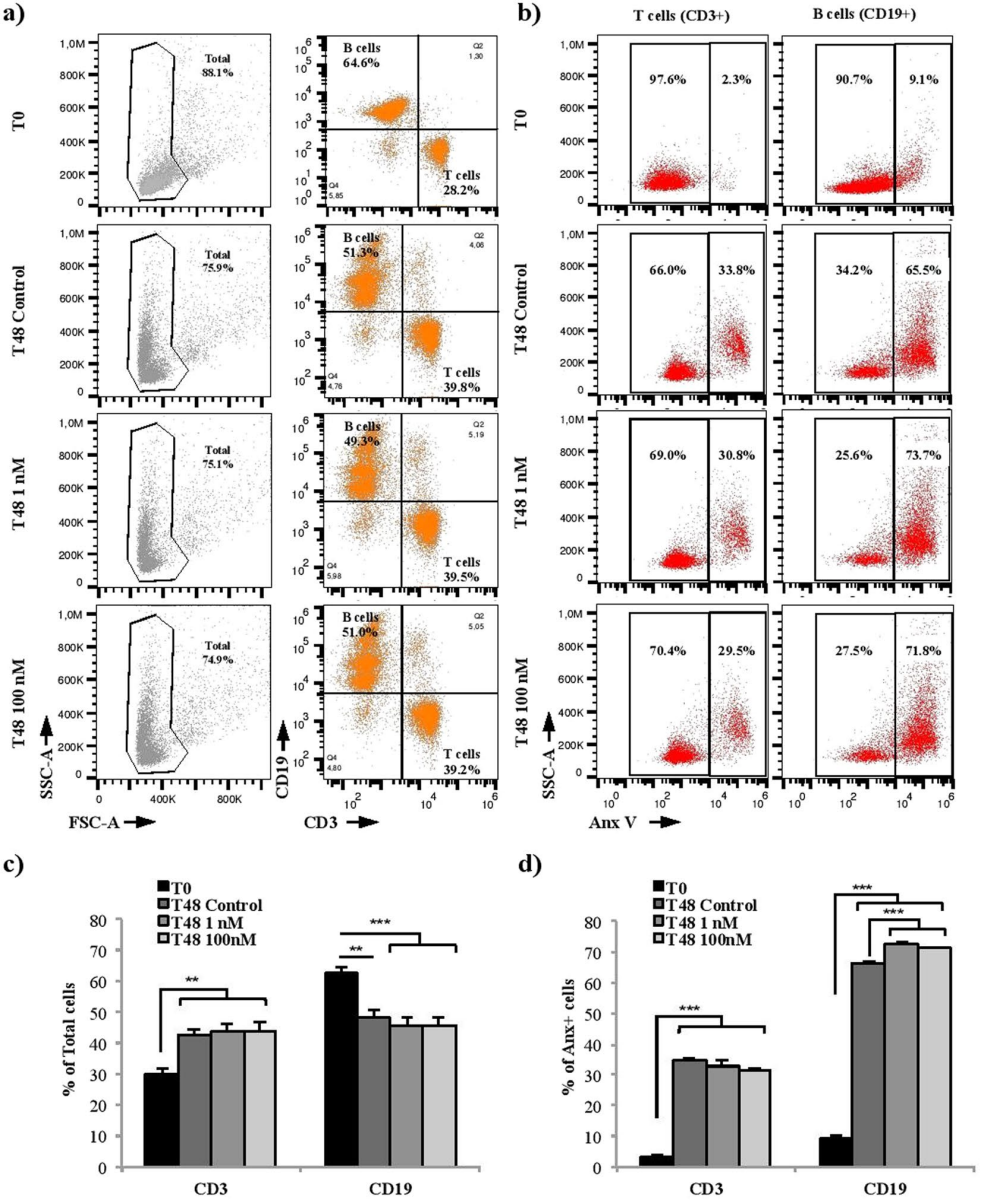
CRH affects murine splenic B lymphocyte viability. Splenocytes cultured for 48 h with CRH 1 or 100 nM were stained with anti-CD3-APC or anti-CD19-PC7 and annexin V-FITC (Anx-V). (**a**) Representative FACS dot-plots gated on total cells. Numbers in the quadrant represent the percentage of B and T cells for each condition. (**c**) Mean percent ± SEM of B and T cells from three independent experiments. (**b**) Representative FACS dot-plots on either T (CD4+) or B (CD19+) cells. Numbers in the quadrant represent the percentage of B or T cells negatively or positively stained with Anx-V for each condition. (**d**) Mean percent ± SEM of gated B (CD19+) and T (CD4+) cells positive for Anx-V from three independent experiments. **p < 0.01, ***p < 0.001.

As it was shown that CRH can induce peripheral blood lymphocyte apoptosis^34^, we next studied the effect of CRH on B cell viability. Splenocytes were cultured with or without CRH 1 or 100 nM for 48 h. Then, T and B cells were labeled with anti-CD3 and anti-CD19 antibodies, respectively, before (T0) and after 48 h of culture (T48). Flow cytometry analyses showed that the percentage of B cells decreased after 48 h of culture with or without CRH while the percentage of the T cells increased (≈ 60% of B cells and 30% of T cells at T0 with a T/B cells ratio of 0.48; ≈50% of B cells and 45% of T cells at T48 with a T/B cells ratio ranging from 0.88 to 0.96) (Fig. 3a and c). Analysis of Annexin-V (Anx-V) staining in both T and B cell populations showed that B cells were more sensitive to apoptosis than T cells (≈10% of B cells and 3% of T cells were Anx-V positive at T0 while 66% of B cells and 35% of T cells were Anx-V positive at T48 without CRH), (Fig. 3b and d). Furthermore, the treatment of splenocytes with CRH significantly increased the rate of Anx-V stained cells only in the B cell population (+6.5% and +5% with CRH 1 nM and 100 nM respectively). These last results demonstrated that, *in vitro*, CRH decreased B cell survival.

### CRHR expression is increased in the spleen after intraperitoneal immunization

**Figure 4:**
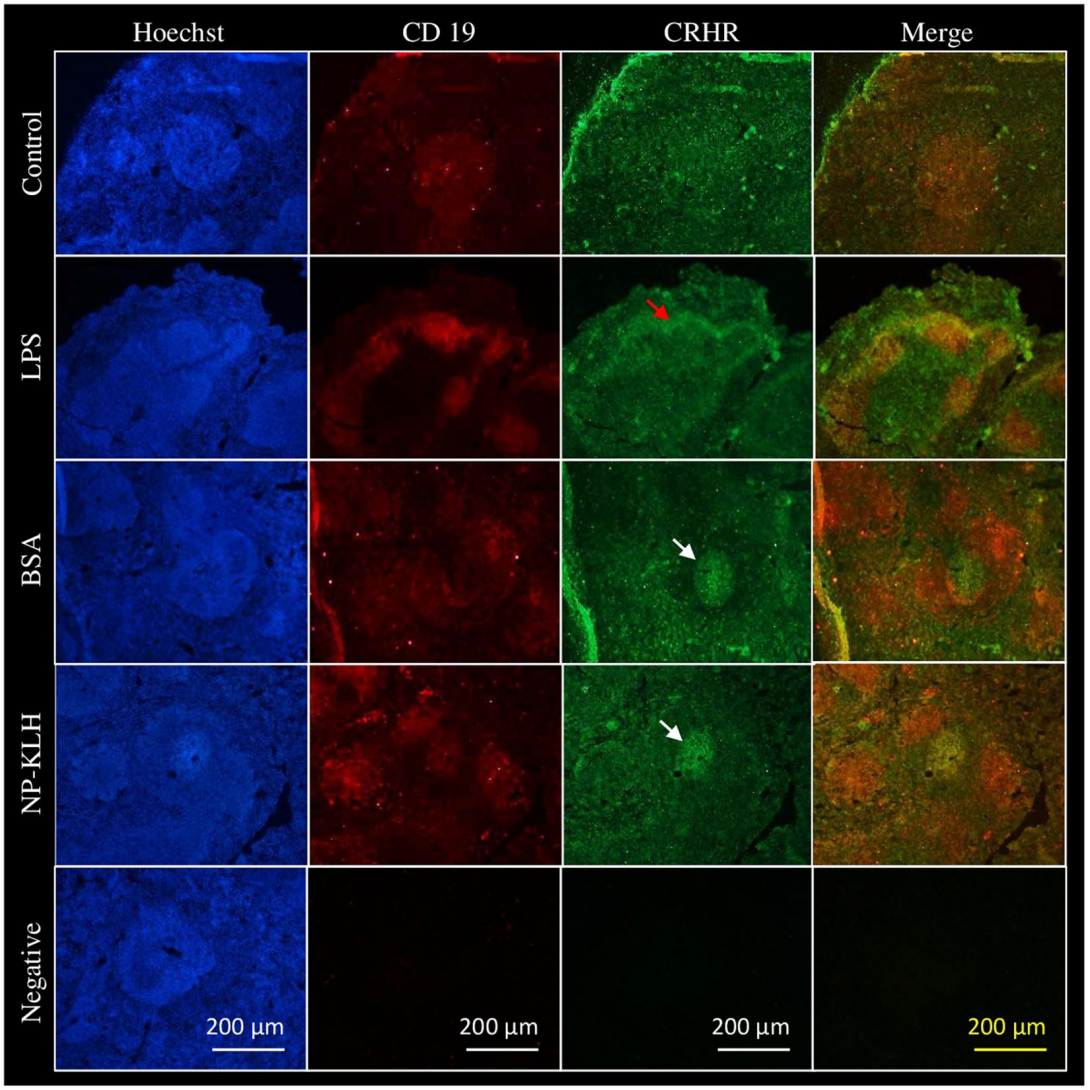
CRHR2 expression in murine spleen after intraperitoneal immunization. Mice were immunized intraperitoneally with T cell-dependent (NP-KLH or BSA) or T cell-independent (LPS) antigens. Ten days later, mice were euthanized and spleens were prepared to perform immunofluorescence experiments with anti-CD19 (red) and anti-CRHR (green) antibodies. Figures representative of three independent experiments. Arrows show localization of CRHR labeling in splenic B cell (CD19^+^) areas. Red arrow indicates CRHR labeling in marginal zone (around B cell follicles) after immunization with T-independent antigen. White arrows indicate CRHR labeling in B cell follicles after immunization with T-dependent antigens.

As reduced humoral immune response and germinal center formation were noted after immunization of CRH-Tg mice, we performed *in vivo* experiments to further understand the function of CRH receptors on splenic B cells. Mice were immunized with two T cell-dependent antigens, BSA (bovine serum albumin) and NP-KLH (4-hydroxy-3-nitrophenylacetyl hapten conjugated to keyhole limpet hemocyanin), or with a T cell-independent antigen, LPS (lipopolysaccharide). Then, immunofluorescence staining was used to assess the expression of CRHR within the spleen (Fig. 4). In non-immunized mice, splenic CRHR labeling showed no precise localization. After immunization with BSA, CRHR staining was increased into B cell areas corresponding to follicles where B cells are known to respond to T cell-dependent antigens and lead to germinal center formation. This result did not depend on antigen composition because immunization with another T cell-dependent antigen, NP-KLH, led to the same CRHR staining localization (white arrows). After immunization with LPS, a more specific CRHR labeling was observed around B cell areas, corresponding to marginal zones (red arrows). In these areas, B cells are known to be activated with T cell-independent antigens. Taken together, these results suggest that whatever the antigen used for immune system activation, the splenic CRHR expression is more specifically localized in B cell areas.

## Discussion

Over the past few years, it has become clear that CRH can exert direct effects on immune tissues, such as the spleen, through the presence of CRH receptors in these tissues. Nevertheless, direct CRH effects on leukocytes, notably on B cells, are not well known yet. Thus, the purpose of our study was to better understand the impact of CRH on B cells.

We first demonstrated by western blotting and immunofluorescence that splenic murine B cells express CRHR and, by sequencing of RT-PCR products, that they express only CRHR2 but not CRHR1. This result is very interesting because it shows for the first time that CRH can act directly on B cells to modulate their physiology during a stress response.

Western blotting experiments performed with the anti-CRHR antibody revealed two bands. In rodents, different CRHR2 mRNAs are generated by alternative splicing leading to two isoforms: CRHR2α and CRH2β^35^. This alternative splicing leads to the deletion of exons 1 and 2 in CRHR2α mRNA and to the deletion of exon 3 in CRHR2β mRNA^36^. The predictive size of these two isoforms is 47 kDa for CRHR2α and 50 kDa for CRHR2β (UniProt; http://www.uniprot.org/uniprot/Q60748). Thus, the two bands observed in our western blotting experiments likely correspond to CRHR2α for the lower band and to CRHR2β for the higher band. This indicates that murine splenic B cells express both isoforms.

CRH is known for having a greater affinity for CRHR1 than for CRHR2 (Kd of 3 nM and 10–40 nM, respectively)^37^. However, different studies have demonstrated that CRH can act *via* its type 2 receptor on neurons or macrophages^38–40^. Once activated, rodent CRHR can activate different phospho-MAP kinases pathways like CREB (cyclic AMP response element-binding), p38 and GSK3β^41–43^. Consequently, we screened different signaling pathways that could be activated by CRH in murine B cells.

Based on the literature, cells were incubated with CRH 100 nM, to mimic high stress levels, because *in vivo* studies estimated that CRH level could rise above 100–200 nM in the hypothalamus and hippocampus of animals placed under stressing conditions^44^. Our results suggest that CRHR2 binding induces the activation of different MAP kinases pathways such as p38 (more precisely p38β and p38δ), GSK3β and CREB. Then, we further investigated the consequences of the presence of CRHR on B cell survival. Indeed, it has been shown that CRHR2 activation induces apoptosis in a murine macrophage cell line (RAW264.7)^43^. Our data show that both CRH 1 (to mimic mild stress levels) and 100 nM lead to an increase of murine splenic T/B cells ratio which is due to a decrease of B but not T cell viability. These results are consistent with *in vitro* studies showing that 1 nM of CRH could affect PC-12 rat adrenal cell line apoptosis and murine oocyte maturation^45,46^. Interestingly, the diminution of B cell survival correlates with proteome phospho-MAPK array results, showing the activation of different MAP kinases pathways such as p38, GSK3β and CREB known to be involved in cell death. Indeed, the increase of the phosphorylation levels of such protein kinases has been associated with apoptosis induction in various cell types such as p38δ in keratinocytes^47^ or GSK3β in neurons^48^ and hepatocytes^49^. In the same manner, in animal models of ischemia-reperfusion injury or neonatal hypoxia-ischemia, GSK3 inhibition protects the heart and the neurons respectively via the diminution of inflammation and apoptosis^50,51^. How CRH specifically impacts B cell survival remains unclear. However, based on our data and on the literature, one could speculate that such early signaling events induced by CRH might lead to the reduced B cell survival.

Our observation of a B cell survival diminution in the presence of CRH is in agreement with a previous study showing an increase of T/B cells ratio in the spleen of mice subjected to a mechanical stress model^52^. Studies performed on CRH-Tg mice revealed the same alteration of T/B cells ratio in the spleen, associated with a B cell maturation blockage and a decrease of germinal center formation after immunization^32,33^. Nevertheless, *in vivo* studies did not dissociate CRH and glucocorticoid action. As CRH overexpression leads to glucocorticoid overproduction and because B cells express glucocorticoid receptors^53^, it is difficult to know if such results are due to a direct impact of CRH on lymphocytes. CRH could potentiate glucocorticoid action and vice-versa. Indeed, in our study, we observed an increase of glucocorticoid receptor transcription levels in murine splenocytes after CRH treatment during 48 h (Supplementary Fig. S2). However, during our *in vitro* studies, there was no glucocorticoid stimulation. Thus, the impact on B cell viability is the direct consequence of CRH action.

Finally, we focused on the *in vivo* role of CRH in splenic B cell maturation and selection. Immunofluorescence staining of spleen sections from mice immunized with different antigens was performed. In non-immunized mice, immunostaining did not show any CRHR specific localization. This result is not surprising because other splenic cells such as macrophages, dendritic cells and T cells are known to express CRHR^54,55^. After immunization with T cell-dependent antigens (BSA or NP-KLH), CRHR staining was more localized in B cell follicles where germinal centers can develop after B cell activation. Germinal center formation, necessary for B cell maturation and selection, takes place in response to T cell-dependent antigens. After immunization with a T cell-independent antigen (LPS), CRHR staining seemed to be situated in marginal zones located around B cell follicles. These observations suggest that, in addition of being a stress hormone able to act directly on B cells, CRH could also play a role in B cell selection and maturation during immunization/infection. This hypothesis could explain why CRH-Tg mice exhibit a diminution of germinal center formation in the spleen, associated with a decrease of immunoglobulin class switching and antibody affinity maturation^32,33^; this last observation having also been done in stressed animals^56^.

## Conclusion and Perspectives

In conclusion, we show for the first time the expression of CRHR2 on splenic murine B cells thereby rendering them more sensitive to death induced by CRH produced during stress responses. Moreover, the increase of CRHR expression in or around B cell follicles after immunization raises the question of the function of CRH in B cell maturation and selection during immune responses. In the future, it would be interesting to determine if peripheral blood B cells also express CRHR2. Indeed, when a tissue is injured, inflammation occurs and permits not only the entry of circulating leukocytes in the stressed tissue but also the increase of HPA axis activity. As this activation leads to an increase of CRH production, it will be important to investigate if this overproduction modulates the physiology of infiltrated leukocytes, especially in the brain where CRH concentration is high. Indeed, neuro-inflammation studies have shown that blood B cells infiltrating the brain could cause deleterious or protective effects depending on the B cell subset^57–59^.

## Methods

### Mice

C57Bl/6J male mice aged 14–18 weeks (Charles River Laboratories, Saint Germain Nuelles, France) were housed in vented animal cabinets (Noroit, Bouaye, France) under controlled temperature (22 °C) and 12 h light-dark cycle with free access to food and water. Animals were treated in accordance with the national legislation. Experimental protocols were approved by the local ethics committee (Comité d’Ethique Lorrain en Matière d’Expérimentation Animale, permit number: CELMEA-2012-008).

### Cell culture and B cell isolation

Mice were anaesthetized with isoflurane (AbbVie, Rungis, France) and euthanized by cervical dislocation. Each spleen was collected and dissociated with 70 μm nylon cell EASYstrainer (Greiner bio-one, Dutscher, Brumath, France). Then, splenic red blood cells were lyzed into 2 mL of 1X RBC lyzis buffer (eBioscience, Affymetrix, Rennes, France) for 2 min at 37 °C and the reaction was stopped by adding 10 mL of RPMI 1640 medium. After centrifugation at 300 g during 5 min, the pellet was suspended in RPMI 1640 medium enriched with 10% heat inactivated fœtal calf serum (Sigma-Aldrich, L’Isles d’Abeau Chesnes, France), 100 U/mL penicillin, 100 μg/mL streptomycin, 10 mM HEPES, 2 mM L-glutamine, 1 mM sodium pyruvate and 1X non-essential amino-acids (all purchased from Sigma-Aldrich). After cell count, splenocytes were cultured at a density of 1 × 10^6^ cells/mL at 37 °C under 5% CO_2_ for 48 h with or without CRH 1 or 100 nM (to mimic mild and high stress levels, respectively), (Bachem, Interchim, Montluçon, France). After incubation with CRH, splenic murine B cells were purified using the MagCellect^TM^ Mouse B cell isolation kit according to manufacturer’s instructions (Stemcell Technologies, Grenoble, France) to perform western blotting experiments. B cell purity was checked by flow cytometry after co-staining with anti CD19-PC7 and anti CD3-APC antibodies. The degree of purity was comprised between 92 and 97%. For RNA extraction followed by RT-PCR and for protein array experiments, B cells were directly purified from mice spleen with the MagCellect^TM^ Mouse B cell isolation kit.

### RT-PCR

Total RNA was extracted from purified splenic B cells using Trizol reagent (Invitrogen, Thermo Fisher Scientific, Villebon sur Yvette, France) and treated with DNase I to remove potential traces of genomic DNA (MBI Fermentas, Thermo Fisher Scientific). For cDNA synthesis, 200 ng of total RNA, 0.5 mM dNTP (Invitrogen) and 50 ng of random primers (Invitrogen) were incubated 5 min at 65 °C. Then, 200 units of M-MLV reverse transcriptase, 40 units of RNaseOUT^TM^ recombinant ribonuclease inhibitor, 1x RT buffer and 10 mM of DTT (all from Invitrogen), in a final volume of 20 μL, were added and incubated 10 min at 25 °C, 50 min at 37 °C and finally 5 min at 70 °C. Sequences of primers used to amplify CRHR1 transcripts were: forward 5′-TGGTCCTGCTGATCAACTTT-3′ and reverse 5′-GTCTCAGATGTGGTGGATGC-3′ (GenBank accession number NM_007762.4). Those used to amplify CRHR2 transcripts were: forward 5′-GGGCATCACCTACATGCTC-3′ and reverse 5′-CAAAGAAACCCTGGAAGGAC-3′ (GenBank accession number AY445512.1). Other pairs of CRHR1 and CRHR2 oligonucleotides (catalog numbers QT00106232 and QT00151543, respectively) were purchased from Qiagen (Courtaboeuf, France) to confirm RT-PCR results. PCR conditions were as follows: activation for 3 min at 95 °C, then 40 cycles of amplification of 15 s at 95 °C and 1 min at 60 °C (Qiagen oligonucleotides) or 61 °C (CRHR1) or 62 °C (CRHR2) followed by 1 min at 95 °C. Each RT-PCR was performed in triplicate.

### Purification and sequencing of amplicons

CRHR1 and CRHR2 PCR products were separated on an agarose gel stained with ethidium bromide. Amplicons were excised and purified using the lysis NucleoSpin Gel and PCR clean-up kit (Macherey-Nagel, Hoerdt, France). Then, purified PCR products were sent for sequencing to GATC Biotech (Mulhouse, France).

### Protein preparation

After CRH stimulation, purified splenic B cells were suspended in NP-40 cell lysis buffer (150 mM sodium chloride, 50 mM Tris pH 8.0, 1% NP-40) and protein extraction was performed in the presence of a complete protease and phosphatase inhibitor mixture (Roche Molecular Biochemicals, Mannheim, Germany). Protein concentrations were determined using a Coomassie protein assay (Bio-Rad, Ivry sur Seine, France).

### Western blotting

Purified B cell protein extracts (20 µg) were subjected to electrophoresis on SDS-polyacrylamide gels (Sigma-Aldrich) that were transferred onto polyvinylidene difluoride (PVDF) membranes (Thermo Fischer Scientific). After blocking, membranes were incubated with an anti-CRHR antibody (diluted 1/1000, BIOSS USA, Interchim). Then, membranes were washed and incubated with an anti-rabbit IgG-peroxidase conjugate (diluted at 1/3000, Jackson ImmunoResearch, Interchim). To quantify the amounts of CRHR relative to the one of GAPDH (glyceraldehyde 3 phosphate dehydrogenase), blots were stripped with stripping buffer (Thermo Fisher Scientific) and reprobed using an anti-GAPDH antibody (diluted /10000, Sigma-Aldrich) followed by an anti-rabbit IgG-peroxidase conjugate. Immunocomplexes were visualized by chemiluminescence (FX7, Vilbert-Lourmat, Marne la Vallée, France) and signals were analyzed by densitometry (ImageJ®, NIH, USA).

### Protein Array

Purified B cells from a pool of three spleens were incubated with or without 100 nM of CRH during 15, 30 or 60 min. Proteins were then extracted and CRH-activated signaling pathways were analyzed using the “proteome profiler human phospho-MAPK array” kit according to manufacturer’s instructions (R&D Systems, Lille, France). 80% of the antibodies present on this array can detect murine proteins according to the manufacturer. Signals were visualized by chemiluminescence (FX7, Vilbert-Lourmat) and analyzed by densitometry (ImageJ®).

### Flow cytometry

Murine splenocytes or purified splenic B cells were stained during 1 h at 4 °C with an anti-CD19 antibody conjugated with PC7 and an anti-CD3 antibody conjugated with APC (eBioscience), each diluted 1/100 in PBS containing 0.5% of BSA (bovine serum albumin, Sigma-Aldrich). A staining with Annexin-V conjugated to FITC (Beckton Dickinson, Le Pont de Claix, France) was performed to evaluate cell viability. Flow cytometry data were collected using the flow cytometer from the Federation of Research FR3209 (Gallios, Beckman Coulter, Villepinte, France) and analyzed using the FlowJo® software (FlowJo LLC, Ashland, USA).

### Splenocyte immunofluorescence

After 48 h of incubation with CRH 1 or 100 nM, 5 × 10^5^ splenocytes/100 μL were deposited on glass slides (StarFrost, Dutscher, Brumath, France) and fixed during 30 min with 4% paraformaldehyde. After washing, cells were permeabilized with PBS containing 0.5% of BSA and 0.2% of Triton X-100 (Sigma-Aldrich). Then, cells were blocked during 1 h with PBS-BSA 0.5%, 0.1% Triton X-100 and 10% of goat serum (Sigma-Aldrich). Afterwards, cells were stained during 1 h with anti-CD19 (1/100, eBioscience) and anti-CRHR (1/50, BIOSS USA, Interchim) antibodies diluted in PBS-BSA 0.5% with 0.1% Triton X-100 and 10% of goat serum. After washing, secondary goat anti-IgG rabbit antibody conjugated to Alexafluor 488 nm and goat anti-IgG rat antibody conjugated to Alexafluor 546 nm (Thermo Fisher Scientific), both diluted 1/250 in PBS-BSA 0.5% containing 0.1% Triton X-100 and 10% of goat serum, were added during 1 h. Glass slides were washed, incubated with Hoechst 33342 during 5 min to stain nuclei (Thermo Fischer Scientific) and mounted in Mowiol medium (Sigma-Aldrich). All steps were performed at room temperature. Signals were detected using an epifluorescence microscope Eclipse 80i (Objective 100x; Nikon, Champigny sur Marne, France).

### Immunization of mice and immunostaining of spleen sections

Mice were immunized intraperitoneally with 10 µg of lipopolysaccharide (LPS, Sigma-Aldrich), 100 µg of BSA or 50 µg of 4-hydroxy-3-nitrophenylacetyl hapten conjugated to keyhole limpet hemocyanin (NP-KLH, LGC BioSearch Technologies, Steinach, Germany). All antigens were mixed with an equal volume of Freund’s adjuvant (final volume of 100 µL; Sigma-Aldrich). Ten days later, animals were euthanized. After fixation by whole body perfusion, spleens were incubated for 24 h in 4% paraformaldehyde followed by 12 h in PBS containing 30% of sucrose. Frozen spleens were cut using a cryostat to obtain 14 μm slice sections that were fixed on polylysine glass slides (Thermo Scientific Fischer). Sections were permeabilized, blocked as described above and stained overnight at 4 °C with anti-CD19 (1/100, eBioscience) and anti-CRHR antibodies (1/50, BIOSS USA, Interchim) diluted in PBS-BSA 0.5% containing 0.1% of Triton X-100 and 5% of goat serum. After washing, secondary antibodies were applied as described above except that incubation lasted 2 h. Hoechst staining was also performed as described above. Signals were detected using an epifluorescence microscope Eclipse 80i (Objective 20x; Nikon).

### Statistical analysis

Homogeneity of variances and normality of distribution were controlled with Levene and Kolmogorov-Smirnov tests, respectively. The level of statistical significance was set at p ≤ 0.05. Intergroup differences were estimated by analysis of variances with one-way ANOVA tests followed by pairwise comparisons with Fisher’s PLSD tests. All statistical tests were performed using the Statview® software (SAS institute Inc, Cary, USA).

## Electronic supplementary material

Supplemental data
