# Supplementary material for: Murine splenic B cells express corticotropin-releasing hormone receptor 2 that affect their viability during a stress response
Source: Sci Rep. 2018 Jan 9;8:143. doi: 10.1038/s41598-017-18401-y (PMC5760685; doi:10.1038/s41598-017-18401-y)
Supplement: Supplementary file 1 — Supplemental data [file 41598_2017_18401_MOESM1_ESM.pdf]

Supplemental information for

Murine splenic B lymphocytes express corticotropin-releasing hormone receptor 2 that affect their viability during a stress response

Guillaume Harlé[1], Sandra Kaminski[1], David Dubayle[2], Jean-Pol Frippiat[1°] and Armelle Ropars[1°]

[1]EA 7300, Stress Immunity Pathogens Laboratory, Faculty of Medicine, Lorraine University, Vandoeuvre-lès-Nancy, France.

[2]CNRS UMR 8119, Centre de neurophysique, physiologie et pathologie, University of Paris Descartes, Paris, France.

°These authors contributed equally to this study.

Correspondence: armelle.ropars@univ-lorraine.fr

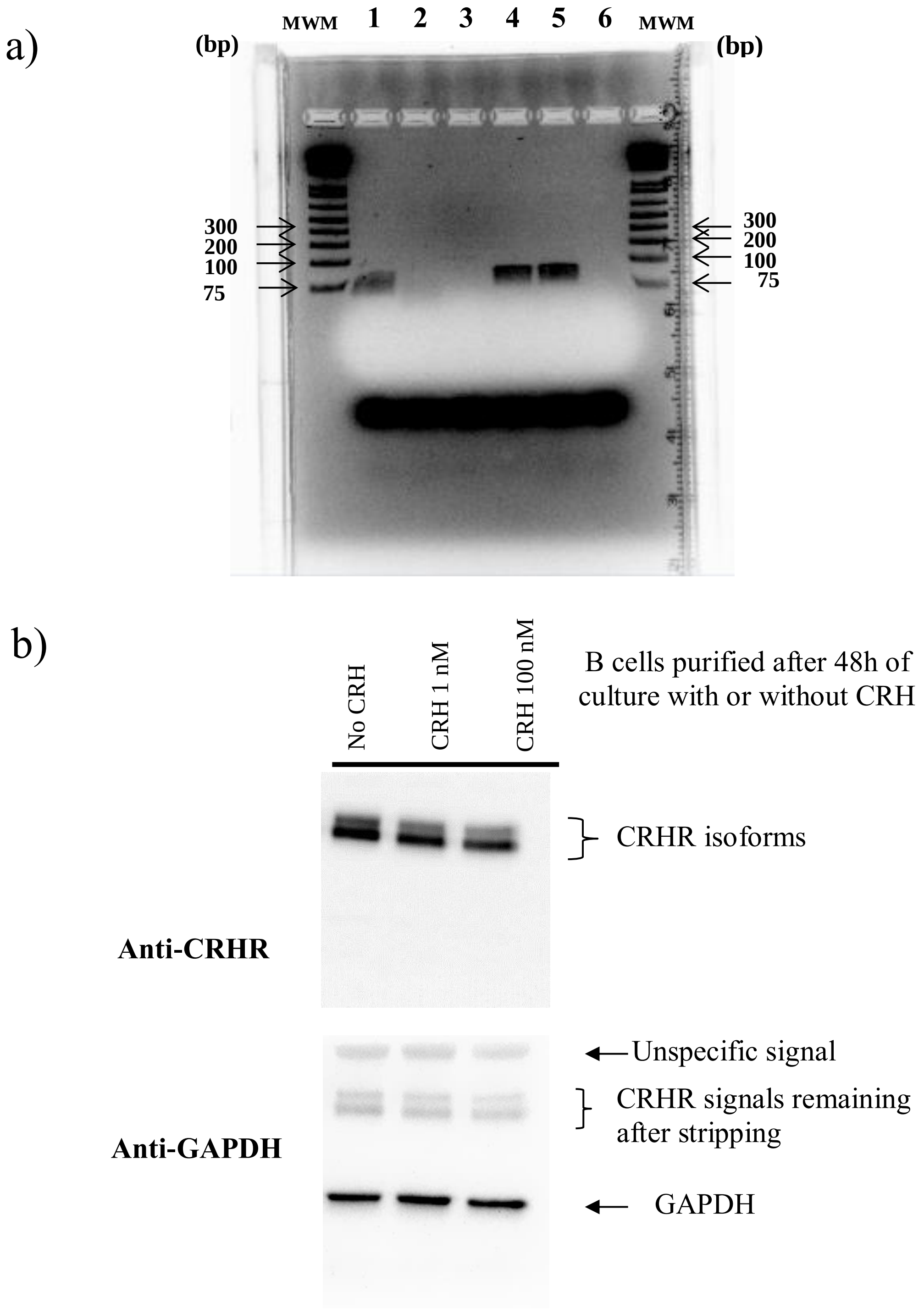

Fig. S1. (a) Full-length picture of the agarose gel presented in Fig. 1a. bp: base pairs. MWM: molecular weight marker. Lanes 1 and 4: CRHR1 and CRHR2 amplification products obtained from hippocampus total RNA (positive controls). Lanes 2 and 5: amplification products obtained from splenic B cell total RNA. Lanes 3 and 6: negative controls (no cDNA). **(b)** Pictures of western blotting results used to construct Fig. 1b. Top panel, bands revealed by the anti-CRHR antibody. Bottom panel, signals after stripping and hybridization with the anti-GAPDH antibody.

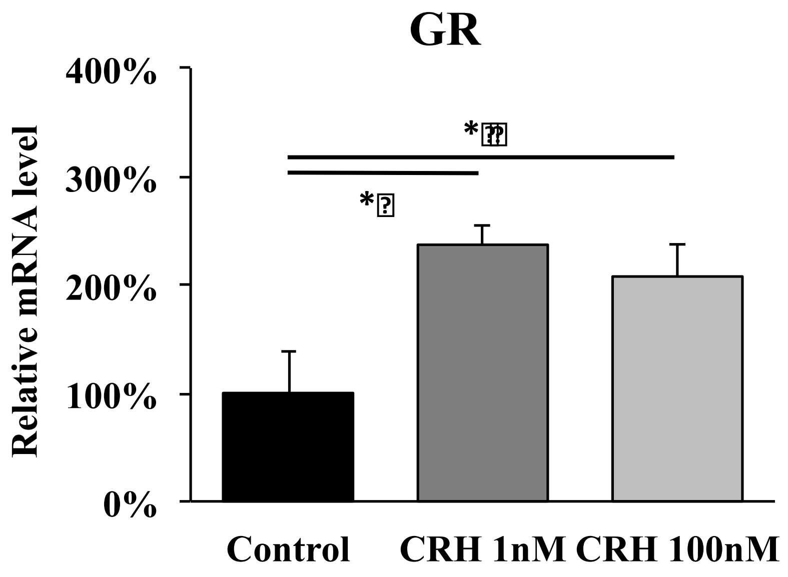

Fig. S2. CRH affects glucocorticoid receptor (GR) mRNA expression level in murine splenocytes. Cells were cultured with CRH 1 or 100 nM for 48h. Total RNA was extracted and reverse-transcribed. GR mRNA expression levels were evaluated by RT-qPCR, as previously described by Huin-Schohn et al.[60], and normalized to three housekeeping transcripts (Ppia, Eef2 and Eif3f). Results are expressed as relative mRNA levels (mean ± SEM) compared to control mRNA levels arbitrarily set at 100%. Figures representative of three independent experiments. * $P<0.05$ vs control.

60. Huin-Schohn, C., Guéguinou, N., Schenten, V., Bascove, M., Gauquelin-Koch, G., Baatout, S., Tschirhart, E. & Frippiat J.-P. Gravity changes during animal development affect IgM heavy-chain transcription and probably lymphopoiesis. *FASEB J.*, **27**, 333-341 (2013).

mRNA (NCBI sequences)	Primer sequences (5'-3')	Annealing temp (°C)	Size (bp)
**Target RNA**			
NR3C1 (GR) (NM_008173.3)	F: CAAGGGTCTGGAGAGGACAA<br>R: TACAGCTTCCACACGTCAGC	61°C	220
**Housekeeping RNA**			
Ppia (NM_008907.1)	F: GTCTCCTTCGAGCTGTTTGC<br>R: GCGTGTAAAGTCACCACCCT	58°C	150
Eef2 (NM_007907.2)	F: GTGGTGGACTGTGTGTCTGG<br>R: CGCTGGAAGGTCTGGTAGAG	58°C	158
Eif3f (NM_025344.2)	F: CATCAAGGCCTATGTCAGCA<br>R: GTGGTGGACTGTGTGTCTGG	61°C	117

Table S1. Primers used for RT-qPCR with NCBI accession number of the sequences used to define them. PCR conditions were as follows: activation for 3 min at 95°C, then 40 cycles of amplification of 15 s at 95°C and 1 min at the annealing temperature indicated in this table for each primer pair followed by 1 min at 95°C. Melting curves were processed from 60°C to 95°C to check the quality of the amplification. Each RT-qPCR was performed in triplicate. F: Forward; R: Reverse.
